# New Uses for the Letheon

**Published:** 1847-04

**Authors:** 


*New uses for the Letheon.*—A correspondent of the "Franco-Americain" writing from Paris, after stating a number of experiments which have been made with the Letheon, preparatory to surgical operations, suggests that this will be found of value in lessening pain of criminals subjected to the guillotine! as also by way of preparation of animals subjected to vivi-sections!

